# Novel Simulation-based Awake Fiberoptic Intubation Curriculum: Pilot Study

**DOI:** 10.5811/westjem.50778

**Published:** 2026-05-19

**Authors:** Daniel Haas, Jenna Fredette, Kathleen A. Murphy, Andrew Deitchman, Jacob Anderson, Maxwell Blodgett

**Affiliations:** * Christiana Care Health System, Department of Emergency Medicine, Newark, Delaware; † WellSpan York Hospital, Department of Emergency Medicine, York, Pennsylvania

## Abstract

**Introduction:**

Awake fiberoptic intubation is a critical skill of emergency physicians in scenarios where rapid sequence intubation may be impossible or catastrophic. While the equipment to perform awake fiberoptic intubation has become more readily available to emergency physicians, inadequate training and lack of confidence are often cited as barriers to performing the procedure. To address this, we created and studied a simulation-based awake fiberoptic intubation curriculum with the goal of improving physicians’ performance of this skill.

**Methods:**

A procedural checklist was developed and iteratively refined among EM, anesthesiology, and critical care physicians. An instructional video was created based on this checklist. Participants viewed the instructional video and underwent supervised deliberate practice on a manikin and bronchoscopy simulator using a rapid-cycle deliberate practice paradigm with 1:1 supervision from an instructor. Comparisons were made between a pre-test and a three-month post-test. We evaluated participants using calculated simulator metrics, the Objective Structured Assessment of Technical Skill global rating scale (GRS), a checklist, and pre- and post-intervention self-assessment.

**Results:**

We collected data from 46 participants. Observed performance using the GRS improved from 22.5 (standard deviation 4.5) to 28.8 (6.3) (*P* < .001). Time from scope insertion to verbalized passage of the endotracheal tube on the simulator decreased from a mean of 147.0 (148.3) seconds to 84.6 (39.1) seconds (*P* = .01). Participants reported improved self-assessed performance compared with others at their stage of training, 3.7 (1.5) to 5.0 (1.2), *P* < .001, and their reported confidence performing the procedure increased from 3.0 (1.5) to 5.1 (1.2), *P* < .001. No significant difference was seen among checklist scores.

**Conclusion:**

In this novel simulation-based awake fiberoptic intubation curriculum, subjective and objective performance improvements were observed at three months. Learners who participated in the course reported feeling more confident and capable of performing awake fiberoptic intubation and being satisfied with the curriculum.

## INTRODUCTION

Airway management is a critical skill for an emergency physician. In a patient with a distorted airway due to trauma, infection, or congenital abnormality, the mechanics of passing an endotracheal tube using a standard laryngoscope may be impossible or unsafe.^1^,^2^ Awake fiberoptic intubation is a technique that has been used in the field of anesthesiology for decades for management of difficult airways.^3^ With the increased availability of disposable bronchoscopes, the necessary equipment for this procedure has become more accessible to emergency physicians. However, the procedure remains infrequently performed in the emergency department (ED).^1^

Awake fiberoptic intubation is often cited as an area of improvement for emergency practice, with 93% of respondents in a recent needs assessment reporting limited opportunities to perform the procedure in clinical practice, and most respondents reported unease performing it.^4^ Furthermore, as a high-acuity, low-occurrence event, hands-on training during patient encounters in the ED is often not sufficient for the physician to become adequately comfortable or proficient with this procedure.^5^

Simulation-based training curricula are well-suited for training in high-acuity, low-occurrence events, given their infrequency in regular clinical practice and high-stakes nature.^6^ Rapid-cycle deliberate practice is an educational framework that uses repeated practice cycles with continuous instructor input.^7^ In rapid-cycle deliberate practice, students are stopped, redirected, and instructed to restart the procedure at any point they fail to adequately complete one of the predefined steps. This technique optimizes trainee time in deliberate practice of challenging steps and minimizes repetition of correctly performed steps, thereby allowing for more rapid acquisition of procedural competency in the simulation setting.^8^ Within the field of emergency medicine (EM), rapid-cycle deliberate practice models have been studied in team-based resuscitation and for instruction of other high-acuity procedures, but to our knowledge the instruction of awake fiberoptic intubation is a novel application of the methodology.^8^–^13^

Currently no standard curriculum exists for the instruction of awake fiberoptic intubation among emergency physicians, and there are no agreed-upon metrics to assess performance. Our goal in this pilot study was to create and evaluate a novel simulation-based rapid-cycle deliberate practice curriculum with the aim of improving awake fiberoptic intubation performance among EM residents and attending physicians. The primary study outcome assessed was improved awake fiberoptic intubation performance on the Objective Structured Assessment of Technical Skill (OSATS) global rating scale (GRS).^14^ The OSATS is among the most widely used tools for evaluating technical skills among surgeons, and validity evidence for it has been collected across a broad range of procedures and contexts.^15^ Secondary outcomes studied included improvements in checklist scores, calculated simulator metrics including decreased total procedure time, and improvements in self-assessed confidence and performance. Additionally, validity evidence was gathered on a novel awake fiberoptic intubation checklist.

**Population Health Research Capsule**

What do we already know about this issue?

*Awake fiberoptic intubation is a potentially life-saving skill for which emergency physicians often feel inadequately trained*.

What was the research question?

*Can a curriculum using a bronchoscopy simulator lead to improved performance of awake fiberoptic intubation?*

What was the major finding of the study?

*Participants demonstrated improvement in observed simulator performance of awake fiberoptic intubation on a global rating scale (p<.001) and in total simulator time (P=.01)*.

How does this improve population health?

*Training in awake fiberoptic intubation can better position physicians to manage airways, which may improve outcomes, particularly among high-risk patients*.

## METHODS

This single-center pilot study took place at a community-academic EM residency training site with an annual volume of over 225,000 patients. The intervention took place between February 2024–January 2025. The study was open to both resident and attending emergency physicians interested in gaining greater proficiency in awake fiberoptic intubation. A convenience sample of volunteer participants was recruited via email and in-person announcements at resident conference. This project was reviewed and approved by the hospital’s institutional review board, and written consent was obtained from all subjects.

We developed a new checklist to evaluate performance of awake fiberoptic intubation among emergency physicians. While prior checklists have been published, our review of the literature found these to be specific to an anesthesia context and lacking in many of the preparatory steps.^16^–^18^ With 95% of respondents in a prior needs assessment of emergency physicians reporting the benefit of a “well-organized approach or algorithm” as a facilitating factor impacting the decision to perform awake fiberoptic intubation, we felt that a more detailed step-by-step checklist would be helpful in instruction and subsequent review of the procedure.^4^

We performed a literature review that informed the creation of a procedural checklist.^19^–^25^ A multidisciplinary team of physicians who were board certified in EM, critical care, and anesthesiology (MB, AD, JA) and had prior experience with awake fiberoptic intubation drafted a checklist and then iteratively refined it in a series of review sessions until consensus was reached (Supplemental Figure 1). The checklist was reviewed by simulation center professionals with training and expertise in the development of procedural checklists. The checklist had 34 maximum points. An instructional video was developed that included relevant anatomy, indications, contraindications, complications, a step-by-step review of the procedure, and troubleshooting steps. The video was approximately 40 minutes in length.

The study took place in a simulation center. On the day of instruction, participants completed a paper pre-course survey, which included participant demographics, relevant prior experience, and self-evaluation of confidence (from “not at all confident” to “very confident” rated on a Likert scale from 1–7) and performance (from “not well at all” to “very well” rated on a Likert scale from 1–7). They were shown the instructional video in situ in the simulation center. Following the instructional video, learners participated in a question-and-answer session followed by hands-on training with 1:1 instructor supervision using a rapid-cycle deliberate practice paradigm. Baseline performance data were collected during the initial training session, after learners viewed the instructional video but before receiving deliberate practice. The same instructor (MB) was used for all participants to minimize variations arising from teaching style. Equipment and patient set-up were performed on a low-fidelity manikin head in a simulated exam room. Insertion and manipulation of the fiberoptic scope was performed using the Simbionix BronchMentor, a bronchoscopy virtual reality trainer (SurgicalScience Sweden AB [publ], Gothenborg, Sweden).

A rapid-cycle deliberate practice approach was used for training during the deliberate practice session wherein learners were given feedback and asked to restart the procedure any time that a checklist step was not performed to mastery level. In alignment with rapid-cycle deliberate practice paradigms, after each step was successfully demonstrated, the procedure was resumed from this step onward, ensuring that every step of the procedure was performed successfully while minimizing unnecessary repetition.

After the initial training session, learners received the procedural checklist and were cued every two weeks to review the procedure through a feedback platform used by the EM residency program, which sent reminders via email and used push notifications. Participants acknowledged within the app when they had completed each biweekly review. Half of participants were randomized to receive mental practice training to supplement their procedural checklist. (The findings of this subgroup analysis will be reported separately.) Data were automatically collected from the bronchoscopy simulator, including total procedural time, percentage of time at mid-lumen, percentage of time with scope-wall contact, attempts to pass when vocal cords closed, and number of times the scope entered the esophagus. Simulator data collection began when the participant inserted the scope into the device, and the case was manually ended as soon as the participant visualized the carina and verbalized placement of the endotracheal tube. In addition to the procedural checklist, the OSATS GRS was used to evaluate procedural performance with a total of 40 possible points.^26^

Participants were reassessed at three months using the same procedural checklist, GRS, and simulator metrics. A post-intervention survey evaluated participant performance, comfort, and satisfaction with the learning experience. A single study author (MB), who was also involved in checklist development, completed all GRS and checklist forms. We compared performance measures and survey responses between the pre-test and post-test at three months. An immediate post-test was not performed. We used an independent two-sample *t*-test to compare the numerical variables, as well as the Likert scale data from the survey. Data are reported as mean (standard deviation). We chose chi-square analysis or the Fisher exact test for intergroup comparisons of demographic categorical variables as appropriate, and categorical variables were expressed as numbers and percentages. *P* values lower than .05 were considered statistically significant. The calculations were performed using statistical software SAS v9.4 (SAS Institute Inc., Cary, NC).

## RESULTS

A total of 51 participants enrolled and 46 completed the study between February 8, 2024–January 23, 2025. Study participant demographics are shown in Table 1. Five participants were enrolled but did not ultimately take part in the study due to scheduling conflicts. Two participants had simulator data excluded from analysis due to technical errors with the simulator.

Objective performance metrics improved between the pre-test and three-month post-test. The primary outcome—observed performance using the GRS—improved from a mean of 22.5 (4.5) to 28.8 (6.3), *P* < .001. Total simulator time, from scope insertion to verbalized passage of the endotracheal tube improved, decreasing from 147.0 (148.3) seconds to 84.6 (39.1) seconds (*P* = .01). No significant change was seen in procedural checklist items completed, which increased from 27.1 (3.5) items to 27.9 (4.0) items (*P* = .27).

Additional improvements were seen in simulator metrics. The proportion of learners who required < 120 seconds for intubation increased from 22/46 (47.83%) to 38/46 (82.61%) (*P* < .001). All the following showed improvements, although none met statistical significance: percentage of time at mid-lumen (66.3 [26.5], 67.1 [25.8]); percentage of time with scope-wall contact (0.4 [1.1], 0.3 [0.9]); number of attempts to pass when vocal cords closed (1.4 [1.6], 1 [0.2]); and number of times the scope entered the esophagus (2.7 [2.7], 1.3 [0.8]).

**Figure 1:**
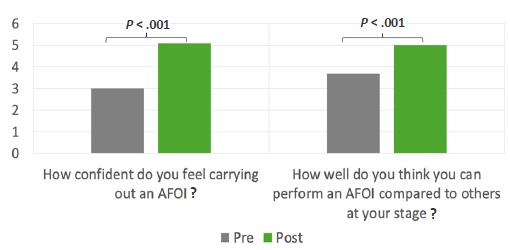
Comparisons of participants’ self-assessed performance between pre- and three-month post-test in a study of a simulation-based awake fiberoptic intubation curriculum. *AFOI*, awake fiberoptic intubation.

Self-assessed performance improved between the pre-test and the three-month post-test as well. Improvement was seen among participants’ rating of “how confident do you feel carrying out an awake fiberoptic intubation”: 3.0 (1.5) to 5.1 (1.2), *P* < .001. Likewise, improvements were seen in response to “how well do you think you can perform an awake fiberoptic intubation compared to others at your stage of training”: 3.7 (1.5) to 5.0 (1.2), *P* < .001 (Figure 1).

Three participants reported having performed an awake fiberoptic intubation in the clinical setting in the period between the pre-test and post-test at three months. Participants responded to biweekly reminders to review the study materials a mean of 4.3 times (2.5) between the initial and post-test sessions. Additional self-reported measures of participation are seen in Table 2. Time between pre-test and post-test sessions was 96.9 (18.2) days.

**Figure 2:**
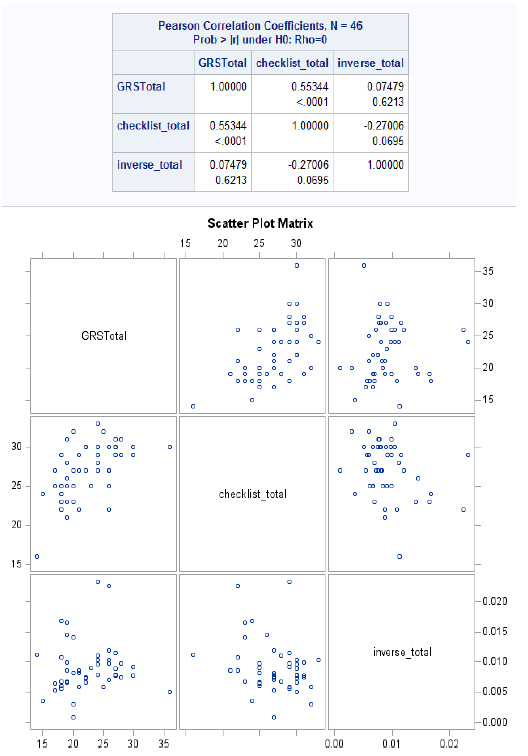
A scatterplot matrix demonstrating pairwise correlations between global rating scale, total checklist items, and the inverse of total procedural time in a study of a simulation-based awake fiberoptic intubation curriculum. *GRS*, global rating scale.

A positive correlation was found between the total checklist score and the GRS, but these did not inversely correlate with total procedure time (Figure 2).

## DISCUSSION

In this single-center pilot study, we developed and implemented a novel simulation-based curriculum to teach awake fiberoptic intubation and evaluate performance among emergency physicians. We demonstrated improvements in both subjective evaluation of performance using a GRS and in objective simulator metrics between a pre-test and a three-month post-test. Survey results suggest participants felt significantly more proficient and confident with the procedure after taking the course.

To our knowledge, no comprehensive curriculum has been previously described for instructing and evaluating awake fiberoptic intubation among emergency physicians. Prior research has shown that brief educational interventions such as ours have the potential to improve skill performance. Naik et al showed that a one-hour training session was sufficient to learn the basics of awake fiberoptic intubation among a group of anesthesiology residents.^18^ Likewise, McCloskey et al showed structured peer coaching can improve comfort with the procedure in a group of emergency faculty.^5^

We found significant improvements in participants’ confidence in their ability to perform awake fiberoptic intubation between a pre-test and a three-month post-test. Emergency physicians generally report limited procedural opportunities and overall low confidence performing awake fiberoptic intubation.^4^ Further, most respondents reported that a lack of confidence was a factor when considering whether to perform the procedure.^4^ The gains in confidence after completing this simulation training may help to overcome this barrier to performing this potentially lifesaving procedure. This curriculum demonstrated consistent improvement in participants’ ability to simulate successful placement of an endotracheal tube positioned above the carina in ≤ 120 seconds between a pre-test and a three-month post-test. A total procedure time of < 2 minutes has been proposed as part of a minimum passing standard in prior studies.^27^,^28^

This study used both a low-fidelity manakin and a virtual reality bronchoscopy task trainer in a simulation lab setting. Simulation-based training models have been shown to consistently increase procedural skill in learners and are well described in the EM literature.^29^ While bronchoscopy task trainers are not commonly used in EM, this simulator equipment has been well described in other procedural specialties and is present in many comprehensive medical simulation settings.^27^,^30^–^34^ Task trainers of this type vary in their capabilities, but many include realistic depictions of anatomy, simulated vital signs, ability to respond to scope maneuvering with haptic feedback, and the ability to collect volumes of quantitative feedback. These task trainers are present in many medical school and hospital simulation labs, as they are used in common simulation courses for endoscopy and bronchoscopy.^35^,^36^

Prior studies have shown that training with bronchoscopy simulators can improve simulated performance of an awake fiberoptic intubation.^31^,^37^ Furthermore, studies have shown that performance improvements of awake fiberoptic intubation in simulated settings directly translate to improved performance in real patients, supporting the notion that the improvements we have described in this study could potentially translate to improved patient outcomes.^18^,^30^,^32^,^38^,^39^

We present validity evidence for this checklist.^40^ A review of the literature to inform checklist development and multispecialty expert review contributed toward content validity. For consistency in response process, the subjects received the same video training and the same author instructed all subjects and answered the Q&A. Additionally, participants were familiarized with equipment prior to data collection. Statistically significant correlations were noted between GRS totals and checklist totals, demonstrating validity evidence of relationship to other variables (Figure 2).

In this study we used a multimodal approach to standardize assessment between learners; this approach included a GRS, a checklist, and objective metrics collected from the bronchoscope simulator. While we noted the absence of a measurable performance increase in checklist scores in our cohort, at the same time we saw improvements in both GRS and simulator metrics. This supports the utility of an evaluation scheme that extends beyond checklists alone. Checklists have been criticized as measures of thoroughness rather than competence, and the use of a GRS may be superior to the use of checklists.^26^,^41^

It is possible that the sample size in our study may not have been sufficient to identify improvement in performance using the checklist alone. It is also possible that the correlation between checklist scores and GRS may be statistically significant but not clinically meaningful considering the absence of corresponding checklist-score improvement. While we saw improvements in GRS scores and in total simulator time, we did not see an inverse correlation between these two variables. This finding may reflect an underpowered sample insufficient to detect a correlation, or it may indicate that these two outcomes capture distinct aspects of competence, as proficiency may occur independently of speed. Further study regarding discriminatory value of items on the checklist may result in a checklist that can better distinguish levels of performance among participants.

Global rating scales have previously been found to have high inter-rater reliability and validity when compared to a performance checklist alone when performing technical skill evaluations.^26^,^42^ The initial study describing the use of the GRS in the context of OSATS demonstrated measures of validity including content validity, inter-rater reliability, internal consistency, correlation between checklist and GRS, and reliable effects of year of training on GRS performance.^14^ The GRS has subsequently been extensively studied, and broad validity evidence has been gathered across contexts.^42^ Prior studies in the anesthesiology literature have described the use of the GRS in the evaluation of trainees performing awake fiberoptic intubation in combination with objective simulator data, although this paradigm is not well described in the EM literature.^18^,^30^,^32^ Correlation among these factors in this study supports the validity of using a multimodal approach for evaluating the performance of this procedure among EM trainees as well.

## LIMITATIONS

This study was limited by its single-center and single-specialty design, use of a single rater, and a small sample size, all of which limit generalizability. The virtual reality bronchoscopy simulator allowed for practice only on standard airway anatomy, which does not represent the range of anatomic and physiologic challenges that would often necessitate performance of an awake fiberoptic intubation in the clinical arena. The nasal approach to awake fiberoptic intubation was not taught due to limitations of the simulator. Future research will be needed to evaluate performance with more challenging simulated anatomy and physiology.

There were significant differences in baseline training and competency among participants, and it is unclear how this may have influenced the results of the study. The design of the study used a single instructor for all deliberate practice to minimize educational variability. Thus, the curriculum will need to be studied among a broader range of instructors to ensure reliability.

The three-month latency period between initial training and post-test includes some degree of natural skill degradation, and the lack of an immediate post-test limits evaluation of training effects absent the skill degradation. We attempted to mitigate this using biweekly reminders for participants to review the procedural checklist. However, there was significant variation in self-reported number of review sessions and total time spent in review among participants, which may have impacted post-test performance. A longer latency period may be necessary to assess more realistic gaps between practice and clinical performance of this infrequently performed procedure.

Lastly, although we attempted to develop a procedural checklist based on best practices from the available literature, the validity evidence for this checklist is limited. Future study will be needed to examine additional validity evidence for this checklist, including collection of internal structure validity evidence assessing measures of test-retest reliability, internal consistency, and item analysis. As a sole study author performed all learner ratings using the checklist, we did not undertake rater training and were unable to collect response-process validity evidence regarding rater comprehension of checklist items or measures of inter-rater reliability. Consequential validity was not examined; further study will be needed to evaluate passing standards as well as implications on learners and patients. Additional study will also be needed to determine standardized score thresholds for a minimum passing standard and the ability of this checklist to discriminate between experts and novices.

## CONCLUSION

This pilot study evaluating a novel simulation-based curriculum to teach awake fiberoptic intubation to emergency physicians demonstrated improvements in performance at three months. High levels of learner satisfaction and confidence were demonstrated, and we observed significant improvements in subjective and objective performance characteristics at a three-month post-test interval. Further study will be needed to collect additional validity evidence on our novel checklist, determine score cutoffs for minimal acceptable performance, evaluate correlation between simulated performance and patient outcomes, and assess application to distorted or more challenging airways.

## Supplementary Information



## Figures and Tables

**Table 1:** Baseline characteristics of participants in a study of a simulation-based awake fiberoptic intubation curriculum.

	n(%)
Level of training	
PGY 1	6 (13.04)
PGY 2	11 (23.91)
PGY 3	9 (19.57)
PGY 4	4 (8.7)
PGY 5	1 (2.17)
Attending	15 (32.61)
Number of times performing awake fiberoptic intubation	
0	28 (60.87)
1–2	14 (30.43)
3–4	2 (4.35)
5–10	1 (2.17)
>10	1 (2.17)
Number of times performing similar procedures	
0	10 (21.74)
1–2	21 (45.65)
3–4	3 (6.52)
5–10	5 (10.87)
>10	7 (15.22)

*PGY*, postgraduate year.

**Table 2:** Participants’ engagement with study material in a study of a simulation-based awake fiberoptic intubation curriculum.

How often did you review the procedure during the study period?	n (%)
Never	3 (6.52)
Less than once a month	5 (10.9)
Once	2 (4.4)
At least once a month	21 (45.7)
Every two weeks	15 (32.6)
How long were your practice sessions?	
< 5 minutes	22 (47.8)
5 – 10 minutes	18 (39.1)
10 – 20 minutes	5 (10.9)
> 20 minutes	1 (2.2)
Did you use the study materials provided?	
No	6 (13.0)
Yes	40 (87.0)
