# Supplementary material for: Novel Simulation-based Awake Fiberoptic Intubation Curriculum: Pilot Study
Source: West J Emerg Med. 2026 May 19;27(3):745–52. doi: 10.5811/westjem.50778 (PMC13246190; doi:10.5811/westjem.50778)
Supplement: Supplementary file 1 [file wjem-27-745-s001.docx]

**Supplemental Figure 1:** Procedural Checklist

Date_______________ Participant ID___________

Circle: Initial training / re-testing

Pre-procedure Preparation

| Preparation of the Patient | Yes | No | Comment |
| --- | --- | --- | --- |
| Explains the procedure to the patient and obtains consent | 1 | 0 |  |
| Performs hand hygiene | 1 | 0 |  |
| Places patient on cardiac monitor | 1 | 0 |  |
| Places pulse oximetry on patient | 1 | 0 |  |
| Positions patient in upright seated position | 1 | 0 |  |

| Preparation of Equipment & Medication | Yes | No | Comment |
| --- | --- | --- | --- |
| Gathers equipment – must include the following  Endoscope  Endotracheal tube  10cc syringe  (2)5cc syringes  Blunt fill needle  Atomizer  Ovassapian airway  Tongue depressor  Quantitative end-tidal CO_2_  Airway box  Bag valve Mask | 1 | 0 |  |
| Gathers medications – must include the following to receive full credit.  Glycopyrrolate  Lidocaine liquid  Lidocaine ointment  An anxiolytic  A Sedative  A paralytic | 1 | 0 |  |
| Ensures the endoscope functions properly (verbalize) | 1 | 0 |  |
| Positions the endotracheal tube on the endoscope | 1 | 0 |  |
| Connects 10cc syringe to the endotracheal tube | 1 | 0 |  |
| Tests the endotracheal tube cuff | 1 | 0 |  |
| Draws up lidocaine into 5cc syringe & connects to working port on endoscope | 1 | 0 |  |
| Draws up second 5cc syringe of lidocaine & connects to atomizer tip | 1 | 0 |  |

| Preparation of Equipment & Medication | Yes | No | Comment |
| --- | --- | --- | --- |
| Administers glycopyrrolate (verbalize) | 1 | 0 |  |
| Dons PPE: gloves and mask with shield | 1 | 0 |  |
| Preoxygenates the patient | 1 | 0 |  |
| Administers lidocaine ointment to the base of the tongue | 1 | 0 |  |
| Administers atomized lidocaine aimed at the following areas.   - Uvula - Soft palate - Posterior pharyngeal wall - Deeper towards larynx | 1 | 0 |  |
| Verbalizes consideration of anxiolytic administration | 1 | 0 |  |
| Administers additional atomized lidocaine to the same areas as indicated above | 1 | 0 |  |
| Places Ovassapian airway in the patient’s mouth | 1 | 0 |  |
| Advances the fiberoptic endoscope along the base of the tongue | 1 | 0 |  |
| Gently advances the scope until right above the vocal cords | 1 | 0 |  |
| Instructs assistant to spray the vocal cords with lidocaine via the working port | 1 | 0 |  |
| Swiftly passes through the vocal cords avoiding direct contact with the cords. | 1 | 0 |  |
| Advances the endoscope until the carina is visualized | 1 | 0 |  |
| Instructs assistant to advance the endotracheal tube (verbalized) | 1 | 0 |  |
| Ensures tip of endotracheal tube is positioned just above the carina | 1 | 0 |  |
| Instructs assistant to inflate endotracheal tube cuff (verbalized) | 1 | 0 |  |
| Connects endotracheal tube to BVM (verbalized) | 1 | 0 |  |
| Checks end-tidal CO_2_ (verbalized) | 1 | 0 |  |
| Administers sedative (verbalized) | 1 | 0 |  |
| Secures the endotracheal tube, or directs respiratory therapy to do so | 1 | 0 |  |
| Orders a confirmatory chest x-ray | 1 | 0 |  |

*cc*, cubic centimeter; *CO_2_*, carbon dioxide; *PPE,* person protective equipment
